# Nutritional behaviour and beliefs of ski-mountaineers: a semi-quantitative and qualitative study

**DOI:** 10.1186/s12970-015-0108-5

**Published:** 2015-12-09

**Authors:** Caroline Praz, Mélanie Granges, Céline Burtin, Bengt Kayser

**Affiliations:** Institute of Sports Sciences and Department of Physiology, University of Lausanne and Institute for Research in Rehabilitation, SuvaCare Rehabilitation Clinic, Sion, Switzerland; Nutrition and Dietetics Department, School of Health, University of Applied Sciences and Arts of Western Switzerland, Geneva, Switzerland; Institute of Sports Sciences and Department of Physiology, University of Lausanne, Géopolis, Campus Dorigny, 1015 Lausanne, Switzerland

**Keywords:** Endurance activity, Food behaviour, Food beliefs, Pre-race nutrition, Energy intake, Ski-mountaineering

## Abstract

**Background:**

Endurance athletes are advised to optimize nutrition prior to races. Little is known about actual athletes’ beliefs, knowledge and nutritional behaviour. We monitored nutritional behaviour of amateur ski-mountaineering athletes during 4 days prior to a major competition to compare it with official recommendations and with the athletes’ beliefs.

**Methods:**

Participants to the two routes of the ’Patrouille des Glaciers’ were recruited (A, 26 km, ascent 1881 m, descent 2341 m, max altitude 3160 m; Z, 53 km, ascent 3994 m, descent 4090 m, max altitude 3650 m). Dietary intake diaries of 40 athletes (21 A, 19 Z) were analysed for energy, carbohydrate, fat, protein and liquid; ten were interviewed about their pre-race nutritional beliefs and behaviour.

**Results:**

Despite belief that pre-race carbohydrate, energy and fluid intake should be increased, energy consumption was 2416 ± 696 (mean ± SD) kcal · day^−1^, 83 ± 17 % of recommended intake, carbohydrate intake was only 46 ± 13 % of minimal recommended (10 g · kg^−1^ · day^−1^) and fluid intake only 2.7 ± 1.0 l · day^−1^.

**Conclusions:**

Our sample of endurance athletes did not comply with pre-race nutritional recommendations despite elementary knowledge and belief to be compliant. In these athletes a clear and reflective nutritional strategy was lacking. This suggests a potential for improving knowledge and compliance with recommendations. Alternatively, some recommendations may be unrealistic.

## Background

For endurance activities with high levels of energy expenditure, energy intake is an important variable to be considered when devising strategies for optimizing performance [1–3]. High exercise intensities over prolonged periods imply high carbohydrate (CHO) and also fat oxidation rates [4]. CHO and fat availability are thus important determinants of energy expenditure and it is paramount that athletes use optimal nutritional strategies, not only to manage intake during races, but also before races, to optimize storage, and after races, to optimize refuelling [2, 3, 5–7]. There exists a plethora of literature on sports nutrition, and several scientific and sports organizations such as the American Dietetic Association (ADA), the Dieticians of Canada (DC) and the American College of Sport Medicine (ACSM) [2, 8], the International Olympic Committee (IOC) [9] and the International Society for Sport Nutrition (ISSN) [10, 11] have published recommendations and guidelines on energy intake before, during and after exercise [3]. Paradoxically, there is relative paucity of literature reporting actual athlete nutritional behaviour [12]. Among athletes, actual nutritional behaviour does not always comply with the official recommendations, because of a lack of knowledge [13], mistaken beliefs, lack of interest or motivation, practical problems, or perhaps intuition [14]. Nutrition knowledge and beliefs can influence food behaviour [15], even if the relationship is not necessarily obvious. Improved nutritional knowledge plays a role in the adoption of healthier food habits [16, 17] and this is likely also the case for sport nutrition. Better insight into actual athlete behaviour and its determinants is of importance for adapting guidelines in view of improving compliance [18, 19].

A particular type endurance sport is ski-mountaineering racing, consisting of climbing uphill on alpine skis with the heals unlocked in special pivoting bindings and adhesive skins applied to the gliding surface, alternated by skiing downhill with the skins removed and the bindings in the locked position. What makes this sport particular is that it combines very strenuous activity in different locomotion modes with exposure to altitude hypoxia and temperature extremes. Especially uphill the exercise intensity is high with a large fraction of time spend around the respiratory compensation threshold [20–22]. The most popular and famous ski-mountaineering races are generally team races that can last from 4 to more than 12 h. We previously quantified energy expenditure during a famous Swiss ski-mountaineering race (‘Patrouille des Glaciers‘) and found that it was very high: more than 20 MJ (4,800 kcal) for the shorter race route (distance: 26 km; altitude differences: +1881 m and −2341 m; maximal altitude: 3160 m) and more than 35 MJ (8,400 kcal) for the longer one (distance: 53 km; altitude differences: +3994 m and−4090 m; maximal altitude: 3650 m) [22].

The goal of the present study was to get a global perspective on pre-race nutritional habits among amateur ski-mountaineers during the 4 days preceding this major multi-hour ski-mountaineering race. Four different aspects were investigated: 1) pre-competition nutritional practice; 2) comparison between practice and recommendations; 3) comparison of food behaviour between participants in longer and shorter races; and 4) knowledge and beliefs about pre-race nutrition.

## Methods

**Fig. 1:**
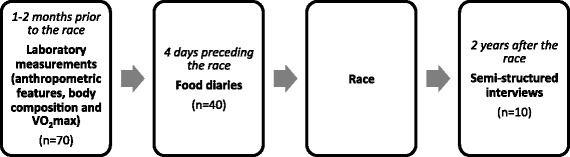
Study flowchart

Seventy participants in two multi-hour ski-mountaineering races were recruited for a study about ski-mountaineering racing (Fig. 1). They were healthy and trained skiers, who took part in one of the two racecourses of the ‘Patrouille des Glaciers’ 2012 [22]. The nutritional part of the study was optional for participants. The Valais cantonal research ethics committee approved the protocol of the study (CCVEM 033/11) and each participant gave informed written consent prior to participation.

### Laboratory measurements

Between 2 and 3 months prior to the race, the subjects came to the laboratory, where anthropometric features, body composition and maximal oxygen uptake (VO_2max_) were determined. Body fat percentage was measured by air displacement plethysmography (BodPod, Cosmed, Italy). The subjects performed a maximal running test on a motorized treadmill (HP Cosmos Pulsar, Germany) to determine VO_2max_. After warm-up (3 min at 5.4 km · h^−1^), speed was set at 7.2 km · h^−1^ and increased by 1.8 km · h^−1^ every 3 min without breaks between stages up to voluntary exhaustion under strong verbal encouragement. The inclination of the treadmill was 0 %. Gas exchange and breathing variables were measured breath-by-breath throughout with a metabolic measurement system (Metalyser, Cortex, Germany). The data obtained during the last 30 s of each step were considered for the analysis. The metabolic system was calibrated prior to each experimental session with a 3 l syringe and gases of known composition.

### The races

The ‘Patrouille des Glaciers’ is the most famous and popular ski-mountaineering race in Switzerland. It is a long duration high altitude team race (teams of three) and consists of two different race routes: race Z from Zermatt to Verbier (distance: 53 km; altitude differences: +3994 m and−4090 m; maximal altitude: 3650 m) and race A from Arolla to Verbier (distance: 26 km; altitude differences: +1881 m and−2341 m; maximal altitude: 3160 m).

### Food intake

We asked the subjects to complete food diaries during the 4 days preceding the race. They had to write down everything they ate or drank with as much detail as possible about the quality and quantity of food items consumed. The subjects received detailed instructions and examples to help them to appropriately complete their diaries. The data were analysed with a nutrient analysis software package (Prodi 5.3, Nutri-Science GmbH, Germany).

Fifty-three journals were collected: 13 were eliminated from analysis, because they were incomplete, unclear or unreadable. 40 journals were analysed: 19 were journals of subjects of race Z (4 women and 15 men, 30 ± 10 years, 176 ± 7 cm, 70 ± 9 kg, 15 ± 5 % of fat mass, VO_2max_: 50 ± 8 ml · kg^−1^ · min^−1^ and 21 of race A (6 women and 15 men, 40 ± 7 years, 176 ± 7 cm, 72 ± 10 kg, 18 ± 8 % fat mass, VO_2max_: 58 ± 8 ml · kg^−1^ · min^−1^). Energy, macronutrients (CHO, fat and proteins) and liquid intakes were analysed.

### Beliefs

Using the diaries, individual semi-structured interviews were held two years later with a pool of 10 subjects of those with a complete diary. These interviews focused on the beliefs about feeding before the race. There were six main questions: 1) tell me more about the importance that you assigned to nutrition during the preparation for the race; 2) what was your food behaviour during the 4 days preceding the race; 3) tell me more about your liquid intake during the 4 days preceding the race; 4) if you were used to consume food supplements, what importance did you assign to these during the 4 days preceding the race; 5) if you were used to consume sports food, which importance did you assign to these during the 4 days preceding the race; and 6) where did you get your information about the nutrition and food strategies you applied? Based on the initial answers to these questions the interviewers asked the participants further questions, to develop and explore details about points that seemed to be especially interesting or needed clarification. Five subjects for each race route were interviewed. We purposely handpicked a diversified sample: three women and seven men, 22 to 56 years, 5 to 37 % of fat mass, VO_2_max from 32 to 67 ml kg^−1^ · min^−1^ (40 ± 10 years, 175 ± 6 cm, 70 ± 11 kg, 16 ± 9 % of fat mass, VO_2_max: 53 ± 10 ml · kg^−1^ · min^−1^).

### Statistical analysis

An ANOVA was used to test whether the four analysed days were similar and to see if pooled mean values could be used for further analysis. For each nutrient the mean and standard deviation values were calculated for the whole population and for the participants in the race Z and the race A separately. T-tests were performed to verify if there were differences between the participants in the shorter and the longer race. Linear regressions were performed to verify associations between sex, body composition or VO_2_max and aspects of nutritional behaviour. Data were analysed with the software Stata (StataCorp, USA). A *p*-value <0.05 was considered significant.

## Results

### Food intake and recommendations

**Fig. 2:**
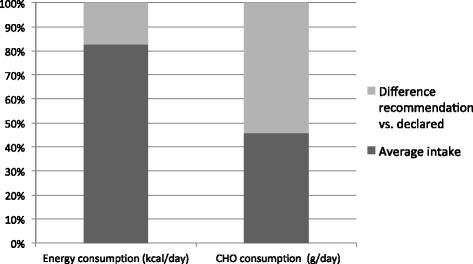
Average percentage of the daily energy intake from CHO, fat and protein. ACSM guidelines for athletes recommend that CHO should represent 50 to 70 % of the total daily energy intake, protein between 10 and 35 % and fat between 20 and 35 % [41]

**Fig. 3:**
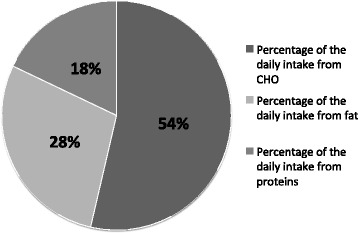
Comparison recommendation-reality for energy and CHO consumption. The entire column represents the recommendation (100 %) and the black part the average declared intake for all 40 subjects (in percentage of the recommendation)

There was no significant difference concerning the main analysed variables (energy, CHO and drinking intake) between the four analysed days, so the data were pooled and the mean values of the 4 days were used for further analysis. The mean energy consumption was 2416 ± 696 kcal · day^−1^. 54 ± 8 % of the daily energy intake was from CHO, 28 ± 6 % from fat and 18 ± 5 % from protein (Table 1, Fig. 1). CHO intake was 46 ± 13 % below the recommended intake levels (10–12 g · body weight (BW) · day^−1^) (Fig. 1). Consequently energy consumption was low too (83 ± 17 % of recommended intake (Harris and Benedict · 1.8 (low exercise intensity, corresponding to a pre-race tapering period) [2]), but the deficit was limited because fat and protein intake partly compensated the lack of CHO intake (Fig. 2). The energy intake through fat was 29 ± 6 % (Fig. 3) of the total daily energy intake (recommendations: between 15 and 30 %) while protein intake was 100 ± 28 % of the minimal recommended intake for athletes in daily life (1.3 g · kg^−1^ BW · day^−1^). The mean liquid intake was 2.7 ± 1.0 l · day^−1^ (slightly above recommendations, 1 ml · kcal^−1^ · day^−1^ in daily life, 2.4 l · day^−1^), partly compliant with the guidelines to already increase the liquid intake before the beginning of a race, without giving a specific amount [23].

**Table 1:** Macronutrients: recommendations and declared pre-race consumption by the participants in races Z and A

	Energy intake (kcal · day^−1^)	Recommended energy intake (kcal · day^−1^)	Protein intake (g · day^−1^)	Recommended protein intake (g · day^−1^)	CHO intake (g · day^−1^)	Recommended CHO intake (g · day^−1^)	Percentage of the total daily energy from fat	Recommended percentage of the total daily energy from fat	Liquid intake (l · day^−1^)
Race Z	2390 ± 569	2892 ± 280	86 ± 22	90 ± 11	315 ± 88	694 ± 86	29 ± 5 %	15-30 %	3.2 ± 1.0
Race A	2428 ± 314	2948 ± 494	96 ± 6	94 ± 15	329 ± 28	721 ± 113	28 ± 7 %	15-30 %	2.3 ± 0.9

One third of the participants took mineral or vitamin supplements. Magnesium was the most commonly used mineral (25 % of the participants) while vitamin supplement intake was also frequent (20 % of the participants). 23 participants (58 %) consumed CHO-rich sports food or drinks, of which 20 % took maltodextrin to increase CHO intake.

There were few differences between the nutritional behaviour of men and women: men consumed more energy from fat (*p* = 0.049) and tended to consume less energy from CHO (*p* = 0.061) than women. A higher body fat percentage was negatively associated with energy intake (*R*^2^ = 0.18, *p* = 0.007), CHO intake (*R*^2^ = 0.29, *p* = 0.001) and liquid intake (*R*^2^ = 0.19, *p* = 0.010) (adjusted for gender) and positively associated with the percentage of the daily energy intake form fat (*R*^2^ = 0.14, *p* = 0.043). A higher VO_2max_ (l · min^−1^) was negatively associated with the lipid intake (*R*^2^ = 0.11, *p* = 0.044).

### Comparison of the food intake of the participants in the shorter and the longer race

No significant differences were noted between macronutrient (CHO, fat and protein) intake of the participants of races A and Z; only liquid intake was higher for the participants in race Z (3.2 ± 1.0 vs. 2.3 ± 0.9 l · day^−1^, *p* = 0.005 (*t*-test, intake adjusted for body mass) (Table 2).

**Table 2:** Supplementation and sports food consumption for each subject from the two race routes

Race Z			Race A		
Subject	Supplementation	Sports food	Subject	Supplementation	Sports food
1	Vitamin C Magnesium	-	20	-	Maltodextrin
2	Magnesium Iron (and vitamins) supplement	Maltodextrin CHO-rich sports drink CHO-rich sports cake Protein shake	21	Magnesium + vitamin C + L-Carnitin	CHO-rich sports drink
3	Homeopathic minerals tablets	Maltodextrin CHO-rich sports drink	22	-	-
4	Magnesium Calcium	CHO and protein-rich sports drink CHO-rich sports drink	23	-	Maltodextrin
5	-	-	24	-	-
6	Multivitamin	CHO-rich sports drink CHO-rich sports cake	25	-	CHO-rich sports drink
7	-	-	26	-	-
8	-	CHO-rich sports drink	27	-	-
9	Multivitamin	Maltodextrin CHO-rich sports drink	28	-	-
10	-	CHO-rich sports cake	29	Magnesium	Maltodextin
11	-	CHO-rich sports drink	30	Rhodiola rosea^a^ Magnesium	-
12	-	CHO-rich isotonic sports drink	31	-	-
13	-	CHO-rich sports drink	32	-	-
14	Mutivitamin (+zinc, calcium, magnesium and guarana)	Maltodextrin CHO-rich sports cake	33	-	-
15	2 Multivitamin and minerals (+caffeine, taurine and guarana) supplement Magnesium	3 Protein shakes Protein bar 2 CHO-rich sports drinks CHO-rich bar	34	-	-
16	Amino acids (aspartate and glutamate) Omega 3	-	35	-	-
17	Vitamin C	Grape sugar	36	-	CHO-rich sports drink
18	-	CHO-rich sports cake	37	-	Maltodextrin CHO-rich sports bar
19	-	2 CHO-rich sports drinks CHO-rich sports cake	38	-	-
			39	-	-
			40	-	2 CHO-rich sports drinks

^a^Rodiola rosea is a medicinal herb containing amino acids, vitamins and minerals

Ten of the 19 (53 %) participants in the race Z used vitamin and/or mineral supplements and 16 (84 %) consumed sports food. Only one person (5 %) used none. For the participants in the race A, three of the 21 (14 %) consumed minerals and/or vitamin supplements and eight (38 %) sports food. Twelve of the 21 participants (57 %) in the race A took neither supplements nor sports food.

### Knowledge and beliefs

All the interviewed subjects indicated that nutrition during the 4 days preceding a long duration ski-mountaineering race is relevant for performance. Six of the 10 interviewed subjects found it important or very important while four found it of little or moderate importance. Three common representations could be highlighted in the interviews: 1) during the 4 days before such a race it is good to eat (lots of) pasta to fill up energy stores (all interviewed subjects); 2) during these days water intake has to be increased (9/10); and 3) it is better to eat white meat than red meat (5/10).

For the comparison of beliefs with practice, as estimated with the food diaries, we found, on the basis of eight main meals over the 4 days analysed for each subject: 1) On average 4.9 ± 1.2 of them included pasta (55 % of meals); these values were 4.4 ± 1.2 meals and 61 %, respectively, for all 40 subjects; 2) The subjects drank 2.2 ± 1.4 l · day^−1^ (2.7 ± 1.0 l · day^−1^ for all 40 participants); 3) For the participants in the race Z, who had dinner just before the race (the participants in the race A started in the morning and had breakfast as the last meal before the race): two of the five interviewed subjects ate white meat during the last dinner (8 of the 19 participants (42 %) who completed the food journal).

In order to enhance performance and to ensure digestive comfort, some other food items were avoided: four athletes avoided or decreased fat food intake (in particular cheese), three athletes spoke about alcohol avoidance, one of the ten took care not to eat too much and one avoided vegetables and salads.

Most of the subjects spoke about food supplements (6/10). Four athletes took magnesium (against muscle cramps (3) or to improve blood flow (1)); one athlete took calcium (also against muscle cramps), one of them spoke about caffeine and one about vitamins. Six indicated that they took sports foods, mostly CHO drinks (4/10), CHO-rich cake (2/10) and maltodextrine (2/10), and protein bars and shakes to preserve muscle mass (1/10).

The most often quoted sources of information about pre-race nutrition were friends and family (4/10), and personal experience and educational background (3/10). Some people got their knowledge from reading (2/10), sports coaches (2/10), sales representatives of sports food brands (2/10), physicians (1/10) or pharmacists (1/10).

The food journals revealed that vitamin and mineral supplement intake, and sports food consumption was higher than what the interviewed subjects said and remembered (Table 2).

## Discussion

Our intention was to explore 4-day pre-race nutritional habits and beliefs among amateur athletes participating to a major multi-hour ski-mountaineering race. The main findings were that 1) the energy and CHO intake was below recommended amounts, while protein and fat intake were close to the recommended intake; 2) the main difference between the participants in the shorter and longer races were liquid, supplement and sports food intakes; and 3) that knowledge and beliefs about pre-race nutrition among these amateur athletes participating to extreme endurance events are insufficient or incorrect.

It is generally believed that high CHO intake to optimize glycogen storage is the most specific and important recommendation for endurance activity to ensure an appropriate energy supply all along a multi hour endurance race [5]. The CHO stores (muscle and liver glycogen) are limited and need to be regularly refuelled. CHO ingestion is thus thought to be very important, whether it is to increase glycogen storage before a workout, during a race to avoid hypoglycaemia and to protect muscle and liver glycogen stores, or after a race to ensure recovery and optimal glycogen resynthesis [6, 7]. Low muscle glycogen stores during exercise, when accompanied by dropping blood glucose levels can cause performance decreases, subjective feelings of low levels of energy, sensation of heavy legs, excess fatigue, loss of concentration, irritability, dizziness and fainting [7, 24]. So it is important to ensure glycogen store fuelling and refuelling and it is better to start a race with full stores [6, 7], especially since the capacity to eat and process food during a race is limited [25]. Current recommendations for CHO storage before an endurance race are 10 to 12 g · kg BW^−1^ · day^−1^, starting 36 to 48 h prior to the race [2, 9, 10].

The average CHO consumption in our population was less than half (46 ± 13 %) of these recommended levels and in fact not even a single participant to our study reached them. Given the belief of the interviewed athletes, that high CHO intake is important prior to such endurance events, this finding is surprising and possibly suggests lack of understanding of the concept of pre-race CHO loading. On the other hand, it may also reflect difficulties in reaching the recommended CHO amounts just by varying the quantities and proportions of the usual dietary components, without using additional specific CHO-rich sports food to allow the athletes reaching CHO and total energy-intake values closer to the guidelines. Our observations lead us to ask the question whether today’s guidelines are adequate for practical use. The observed average CHO consumption in our sample was so far from the recommendations that it suggests that it might have been too difficult for our participants to reach the recommendations by consuming twice as much CHO as they did, even if it were only during the last 24 to 36 h and not during the full 4 days we looked at.

For the other macronutrients: fat and proteins, there are no specific pre-race recommendations for endurance activities, but there are guidelines for athletes in daily life. Endurance athletes are advised to consume between 1.2 and 1.8 g · kg BW ^-1^ · day^−1^ of protein [8, 9] and daily fat intake should amount to 20 to 35 % of total energy intake [2, 8–10]. For these nutrients the intakes of the subjects complied with the guidelines.

Fluid and electrolyte intake are thought to be important for performance because dehydration, when exceeding 2-3 % of body mass, may cause performance impairment [2], even though there is on-going debate on this topic [26]. So it is important to start races in euhydration and to drink enough and frequently during races [8]. Since we only have information about pre-race behaviour and have no data on habitual fluid intake in our subjects we do not know if, according to the recommendations, they increased their fluid intake prior to the race. Intake was on average 2.7 l · day^−1^, which is slightly above the recommendations for habitual daily intake (2.4 l · day^−1^) [23]. Also in this case the beliefs of the interviewed athletes contrast with their behaviour since 9 out of 10 mentioned the importance of increasing liquid intake in the last days prior to a major race but only drank an average 2.2 l · day^−1^.

Although several studies showed that vitamin and/or mineral supplementation does not improve performance during anaerobic [27], strength [28], endurance [28, 29] or ultra-endurance [30] exercise, if the daily diet is adequate [31], supplement intake remains widespread among athletes [30, 32, 33]. In our population, a third of the participants took such supplements. The recommendation is to abstain from vitamin or mineral supplementation if the athlete eats enough and a wide variety of food and in the absence of a known deficit [3]. Additionally, it is important to be careful and to estimate the safety, the efficacy, the potency and the legality of a supplement before taking it [2, 8–10]. Besides supplements, special sports food consumption was also widespread. Endurance athletes mostly consume special CHO drinks, gels or bars, which can help reaching the recommendations for energy and CHO intake [18, 34]. But in spite of 58 % of the 40 participants using CHO-rich sports food or drinks, their CHO intake remained far below the recommendations.

Digestive comfort is another key variable during endurance events such as multi-hour ski-mountaineering races [35, 36]. Digestive discomfort and gastrointestinal distress like cramps, nausea, vomiting, bloating, and diarrhoea are frequently reported during ultra-endurance activities, particularly during ultra-marathon running [35–37]. To avoid gastrointestinal distress during racing, athletes are advised to avoid dehydration, high-fibre food intake, and hypertonic beverages, and to practice their planned race nutrition strategies before the actual race [36–38]. It is difficult to say from the food diaries which food items were specifically avoided during these 4 days, because we do not know the habitual diet of the participants, but from the interviews it seemed that mostly red meat and other fatty food items were avoided.

In general, the athletes seemed to be well aware of the importance of pre-race nutrition for performance. The participants in the longer race Z placed a more particular emphasis on this topic than the participants to the shorter race A. They also trained more, used lighter racing gear (12.4 ± 2.5 kg vs. 15.4 ± 3.2 kg) and their supplement and sports food intake was higher. The participants in the longer Z race were likely more ambitious and experienced athletes, who tried noticeably harder to optimize everything for the race. Our finding of lower lipid consumption by participants with a higher VO_2_max and/or lower body fat percentage, as well as the higher energy, CHO and liquid consumption of participants with lower body fat percentage also seem in accordance with this contention.

In spite of the declared importance of adequate nutrition in preparation of an endurance event, the knowledge on this topic was approximate. Half of the interviewed participants confused supplementation with sports food; all the participants believed that it is good for them to eat a lot of pasta during the 4 days preceding the race, but rarely spoke about CHO and other CHO sources. Moreover, it seemed to be difficult for them to explain why they choose some food items and avoided others. The overall impression was that it rather reflected beliefs than knowledge [15].

The finding in the interviewed athletes that they underestimated their sports food and supplement intake compared to the intake reported in the food journals is probably partly due to the long period of time between the real intake and the interviews. But another important reason for this difference is probably the fact that the athletes did not follow a clear strategy based on solid knowledge. This lack of a clear knowledge and strategy can be a reason for the far too low energy and CHO consumption.

### Limitations

Our results should be interpreted taking into account some study limitations. Despite participation of 70 subjects to the study we obtained only 40 complete food intake diaries that could be analysed of which 25 % was discussed in detail during the face-to-face semi-structured interviews. The number of observations is therefore limited and not necessarily representative for all amateur athletes participating to the ‘Patrouille des Glaciers’ and similar extreme endurance events. Also, the interviews were performed two years after the race and obviously some detail may have been lost. However, it is unlikely that the major individual beliefs changed over these two years [39]. The overall strategy, grounded in the personal beliefs was probably close to that of race day. Finally, food diaries are known to be unreliable with frequent under and ill reporting of intake [40], so that our results should be interpreted as semi-quantitative.

## Conclusions

This study about pre-competition nutritional habits and beliefs of amateur athletes before a long duration ski-mountaineering race showed that: although most of the athletes seemed aware of the importance of nutrition for endurance sports and specifically before a major race, their knowledge was approximate and a clear sound nutritional strategy was missing. Their average CHO intake represented less than half of the recommended intake, while energy intake was on average 17 % too low. There was no significant difference in the energy and macronutrient intake of the participants in the longer and the shorter race, but participants in the longer race drank significantly more, used more mineral and vitamin supplements and more sports food and drinks.

Taking into consideration all of these issues, some recommendations can be formulated for pre-race nutrition:

1. The athletes should be better informed about nutrition to allow them developing an evidence-based conscious and reflective feeding strategy.
2. A high priority should be given to CHO intake. The amount should be increased with traditional food (pasta, rice, bread, etc.), completed with CHO-rich sports food and drinks.
3. The chosen pre-race nutritional strategy should be experimented before that prior to the actual race (e.g. before a hard training period or a less important competition) to ensure that it does not cause gastrointestinal distress.
